# 

**Published:** 1825-06

**Authors:** 


### MONTHLY CATALOGUE OF MEDICAL BOOKS.

*** *It having been hinted to us that gentlemen, sending copies of their Works, prefer having their titles given at length, it is our intention, in future, to comply with this suggestion: but it is to be observed, that no books can be entered on this List except those sent to us for the purpose;—finding, in the lists hitherto transmitted, that the names of works have frequently been given as published, which have not appeared for weeks, or even months, after.*

An Account of the Disease lately prevalent at the General Penitentiary. By P. MERE LATHAM, M.D. Fellow of the Royal College of Physicians, and Physician to St. Bartholomew's Hospital.—pp. 286. London, 1825.

Observations on the Cholera Morbus of India. A Letter addressed to the Honourable the Court of Directors of the East-India Company. By WHITELAW AINSLIE, M.D. M.R.A.S. late of the Medical Staff of Southern India.—pp. 90. London, 1825.

A Probationary Essay on the Extraction of Calculi from the Urinary Bladder, containing some Account of certain Methods that have been recently proposed; submitted, by authority of the President and his Council, to the Examination of the Royal College of Surgeons of Edinburgh, when Candidate for admission into their Body, in conformity to their Regulations respecting the admission of Ordinary Fellows. By WM. THOMSON. March 1825.—pp. 50. Edinburgh, 1825.

A Conspectus of Prescriptions in Medicine, Surgery, and Midwifery; with useful Memoranda of the Symptoms, Diagnosis, and Treatment of Diseases and Injuries, containing upwards of a thousand modern Formulæ, including the new French Medicines, and arranged Tables of Doses. Selected from the highest Professional Authorities; intended as a Remembrancer for General Practitioners. —pp. 174. London, 1825.

An Elementary Description of the Anatomy and Physiology of the Brain, Viscera of the Thorax, Abdomen, &c. with corresponding Questions: designed more particularly for the use of Gentlemen preparing for Examination at Apothecaries' Hall; also for junior Students. By a Licentiate of the Society of Apothecaries.—pp. 57. London, 1825.

A Popular Explanation of the Elements and General Laws of Chemistry. By WALTER WELDON.—pp. 622. London, 1825.

Novus Thesaurus Semiolices Pathologicæ quem Collegit atque edidit MAURITIUS HOOPER, Med. Chir. atque Philosoph. Doctor, &c. &c. Vol. I.—pp. 470. Lipsiæ, 1825.

Tractatus de Vulneribus Pectoris Penetrantillus. Auctore CAROLO MAYER, &c. &c. &c. pars prima; accedit Tabula Lithographica.—pp. 183. Petropoli, 1823.

Illustrations of Acoustic Surgery. By THOMAS BUCHANAN, C.M. Licentiate of the University of Glasgow, Member of the Royal Medical Society, &c. &c. and Surgeon to the Hull Dispensary for Diseases of the Eye and Ear; and Author of the "Guide to Acoustic Surgery."—London, 1825.

An Essay on Tetanus, founded on Cases and Experiments. By JOSEPH SWAN, Member of the Royal College of Surgeons, and Surgeon to the Lincoln County Hospital.—London, 1825.

Parental Guide, during the Progress of the Cow-Pock Disease. By JOSEPH MORLEY, Surgeon, of Wellingore, in the County of Lincoln; Fellow of the Royal College of Surgeons, London.—pp. 40. Lincoln, 1825.

Practical Directions for Preserving the Teeth; with an Account of the most modern and improved Methods of supplying their Loss; and a Notice of an improved Artificial Palate, invented by the Author. Illustrated by Plates. By ANDREW CLARK, Dentist.—pp. 96. London, 1825.

Observations on the Extraction of Diseased Ovaria; illustrated by coloured Plates after Nature. By JOHN LIZARS, Surgeon, Author of the "System of Anatomical Plates," &c.—pp. 24. Edinburgh, 1825.

### NOTICE TO CORRESPONDENTS.

*We have to announce Communications from* Dr. H. Davies, Dr. Webster, Mr. Money, Dr. Kinneir, Dr. Gibson, *and* Mr. Goodeve.

Mr. Watkinson's *and* Mr. Blackett's *in our next: they have been postponed on account of the Engravings required.*

*The continuation of* Mr. Jones's *Paper did not come to hand till the Original Department was made up: it is, therefore, unavoidably delayed till next month.*

☞ *We have been again obliged to exceed our prescribed limits, having added four extra pages to the present Number.*

### INDEX

OF

THE FIFTY-THIRD VOLUME

OF THE

### London Medical and Physical Journal.

PAGE

ABSCESS of the Liver, case of . 43
Absorption, experiments respecting 9
Abdomen, bloody Tumor in the . 529
Acupuncturation, as a cure for Ophthalmia . . . 437
————, death from . 531
————, on the galvanic phenomena of . . . 434
————, accidents from . 260
Action, Mercurial, on the . . 178
Acid in the Stomach . 13, 530
Active principle of Sarsaparilla . 343
Affection of the Brain, chronic . 164
Amputation, remarks on . . 31
———— of the Hip-joint, case of 21
Anatomy, comparative, of the Brain 1
————, pathological, of the Peritoneum . . . 11
Analysis of Medical Evidence . 213
Anomalous Gout . . . 13
Antimony in Dyspepsia . . 529
Appearances, unusual, in the Blood 435
Arteries, on the muscularity of . 87
Auricle of the Heart, rupture of 193

Bark, Pomegranate, in Tænia . 165
——, on the mode of administering 295
Belladonna, on the active principle of . . . . . 169
Birds, on the internal Ear of . 254
Blood-letting, remarks on . 15
Blood, unusual appearances in . 435
——, the rust of, mode of detecting 531
Bones of the Cranium, unnatural growth of the . . . 529
Brain, comparative anatomy of . 1
——, chronic affection of . 164
——, on the enlargement of . 256

Calculi, instrument for breaking . 32
Carotid Artery, instrument to compress . . . . . 383
Case of Amputation at the Hip . 21
——— Abscess of the Liver . 43
——— of Pregnancy with Twins 121
——— Irritative Fever . . 162

PAGE

Case of Purpura Hæmorrhagica . 175
——— punctured Wound . 180
——— Rupture of the Auricle of the Heart . . . 193
——— diseased Spinal Marrow . 257
——— Perforation of the Stomach 287
Cases of Tic Douloreux . . 474
——— Disease of the Heart . 477
Child, a nail swallowed by a . 480
Chemical properties of Mercury, on the . . . . 127
Child crying in Utero . . 260
Children, on the Convulsions of 39, 284
Cicatrisation of the Heart . 259
Cinchona, new species of . . 436
Cochlea of the internal Ear of Birds 254
Colocynth, on the active principle of 170
Contraction, Muscular, on . 10
Contagious property of the Varioloid Disease . . . 461
Contagion, observations on, 263, 351, 448
Couching among the Hindoos, mode of performing . . . 343
Croton Oil, use of, in Inflammation 436
Crystalline Lens, on the reproduction of . . . . 529
Cranium, unnatural growth of the bones of . . . ib.

Death from Acupuncturation . 531
Deformities of the Pelvis on . 531
Depression and fracture of the Frontal Bone . . . 182
Development of the Heart in the Fœtus . . . . 11
Diabetic patients, sugar in the blood of . . . . 163
Dissection, wounds received in, method of preventing . . 530
Diseases, new system of treating . 14
Disappearance of the Mammæ . 437
Dyspepsia, Ipecacuanha in . 529
————, Antimony in . . ib.

Effects of a hot bath . . 165

PAGE
Enlargement of the Brain, on the 256
Ergot, on the employment of . 18
Excision of the lower Jaw, case of 26
Experiments on Absorption . 9
Extirpation of a large Tumor . 25
——— the Ovaria . . 27
Extract of Nux Vomica in Paralysis 341
Extracts from the Medical Jurisprudence . . . . 393
Euphorbia Lathyris, utility of the oil of . . . . . 530

Fœtus, development of the Heart in 11
Fever, on the pathology of 108, 289
———, utility of fresh vegetables in 165
Fracture-cradle for the lower Extremity . . . . . 342
Frontal Bone, fracture and depression of . . . . 182

Gastrotomy, remarks on . . 191
Generation, remarks on . . 10
Galvanic phenomena of Acupuncturation . . . . . 434
Goitre, remarks on . . 49
Gonorrhœa and Syphilis, use of Iodine in . . . 19
Gout, on the anomalous . . 13
———, on the pathology of . 385

Heart, cicatrisation of the . 259
———, diseased, case of . 477
Hernia, on the treatment of . 54
Hindoos, mode of Couching used by them . . . . . 343
Hot bath, effects of . . 165
Humoral Pathology, on the . 364

Iodine, its use in Gonorrhœa and Syphilis . . . . . 19
Inclined plane, on the use of . 481
Inflammation of the Nerves, on . 11
———, Tartar Emetic as a cure for . . . . . 17
———, use of Oil of Croton in . . . . . 436
Instrument for breaking Calculi . 32
——— compressing the Carotid Artery . . . 380
Internal Ear of Birds, on the Cochlea of . . . 254
Intestines, strangulation of the . 435
Ipecacuanha in Dyspepsia . 529
Irritability, puerperal, remarks on, 116, 381
Irritative Fever, case of . 162, 438

Lacteal system, on the . . 9
Lobes of the Brain and Tongue, connexion between . . 528
Lower Jaw, excision of . . 26

PAGE
Lower Extremity, fracture-cradle for . . . . . 342
Lymphatic system, on the . 9
——— vessel, opening in the Vena Cava . . . 255

Mammæ, disappearance of . 437
Medical Topography of Malta, on 194
——— Evidence, analysis of . 213
——— Jurisprudence, extracts from . . . . . 393
Membrana Tympani, uses of . 8
Mercury, on the chemical properties of . . . . 127
——— in the Urine . . 164
Mercurial action, on the . . 178
Midwifery, remarks on . . 170
Method of preserving dead bodies 528
Mode of administering the Bark . 295
Morphia, on the preparation of . 168
Motions of the Tongue . . 528
Muscular contraction, on . . 10
Muscularity of Arteries, on the . 87

Nail swallowed by a Child . 480
Nerves, on the inflammation of . 11
——— of Sense, on the sensibility of . . . . . 434
Neuralgia, remarks on . . 124
New species of Cinchona . . 436
——— system of treating diseases . 14
Nævi, remarks on . . 166
Nux Vomica, on the extract of, in Paralysis . . . 341

Observations on the Convulsions of Children . . . . 39
——— Stomach Syringe 206
——— Contagion, 263, 351, 448
——— the Operation for Strangulated Hernia . . 467
Oil of Turpentine, its use in Rheumatism . . . . 18
——— the Euphorbia Lathyris, utility of . - . . 530
Olfactory Nerves, on the . . 528
Optic Nerves, semi-decussation of 48
Ophthalmia, on the treatment of 122
———, Acupuncturation as a cure in . . . . 437
Ovaria, on the extirpation of . 27

Pathology, on the Humoral . 364
——— of Fever, on the . 108
——— of Gout, on the . 385
Paralysis, on the extract of Nux Vomica in . . . 341
Pelvis, on deformities of . . 531
Peritoneum, on the pathological Anatomy of . . . . 11

PAGE
Pigmentum Nigrum, on the . 8
Plane, on the use of the inclined 481
Pomegranate Bark in Tænia . 165
Preserving dead bodies, method of 528
Pregnancy with Twins, case of . 121
Preparation of Morphia, on the . 168
Proceedings in the University of Edinburgh . . . 80
Principle, on the active, of Belladonna . . . . 169
——— Colocynth 170
Puerperal Irritability, remarks on 116
Purpura Hæmorrhagica, case of 175
Pustules under the Tongue in Hydrophobia . . . 163
Purgative medicine, on the use and abuse of . . . 185

Remarks on Amputation . . 31
——— Blood-letting . . 15
——— Gastrotomy . . 191
——— Goitre . . 49
——— Lateral Curvature of the Spine . . . 167
——— Nævi . . . 166
——— Neuralgia . . 124
——— Midwifery . . 170
——— Snuff-taking . . 166
——— Spinal Diseases . 443
——— Sympathy . . 204
Removal of great part of the Scapula . . . . . 26
Reproduction of the Crystalline Lens, on the . . . 529
Rheumatism, on the use of Oil of Turpentine in . . . 18
Rust of blood, mode of detecting 531
Rupture of the Spinal Marrow . 435
——— Auricle of the Heart 193

Sarsaparilla, on the active principle of . . . 343
Scapula, removal of a portion of . 26
Semi-decussation of the Optic Nerves 48
Sensibility of the Nerves of Sense, on the . . . . 434
Smell, sense of, independent of the Olfactory Nerves . . 528
Snuff-taking, remarks on . . 166
Spine, remarks on the Lateral Curvature of . . . 167

PAGE
Spinal diseases, remarks on . 443
——— Marrow, case of diseased . 257
———, rupture of . 435
Stomach, acid in the . 13, 530
——— Syringe, observations on 208
———, perforation of the, case of 287
Strangulated Hernia, observations on the operation for . . 467
Strangulation of the Intestines . 435
Sugar in the blood of diabetic patients . . . 163
Sympathy, remarks on . . 204
System, Lacteal, on the . . 9
———, Lymphatic, on the . ib.

Table of new Vegetable Principles 38
Tartar Emetic in Inflammation . 17
Tænia, Pomegranate Bark in . 165
Tic Douloreux, cases of . . 474
Tongue, Pustules under the, in Hydrophobia . . . 163
Topography, Medical, of Malta . 194
Treatment of Hernia, on the . 54
——— Ophthalmia, on the 122
——— Wounds received in Dissection . . . 364
Tumor, extirpation of a large . 25
———, bloody, in the Abdomen . 529

Varioloid disease, on the contagious property of . . . 461
Vena Cava, lymphatic vessel opening in . . . . 255
Vegetables, fresh, utility of, in Fever . . . . 165
Vegetable principles, new, Table of 38

University of Edinburgh, proceedings in . . . . 80
Urine, Mercury in the . . 164
Use and abuse of purgative medicine, on . . - 185
Uses of the Membrana Tympani . 8
Uterus, Child crying in the . 260
Utility of the Oil of Euphorbia Lathyris . . . . 530

Wound, punctured, case of . 180
Wounds received in Dissection, treatment of . . 369, 530

### REVIEWS.

### DOMESTIC.

PAGE

Lectures on Digestion and Diet. By CHARLES TURNER THACKRAH, Member of the Royal College of Surgeons of London, of the Societé de Medecine pratique de Paris . . . . . . . . 56

Report of the Epidemic Cholera, as it has appeared in the Territories subject to the Presidency of Fort St. George. Drawn up by order of the Government, under the superintendance of the Medical Board. By WILLIAM SCOT, Surgeon, and Secretary to the Board. With an Appendix, containing the Sick Returns, &c. &c. . . . . 68, 149

Clinical Report on Dropsies; with Observations explanatory of their Pathology and Therapeutics. With an Appendix, on the Theory and Treatment of Organic Diseases in general. By ROBERT VENABLES, Bachelor in Medicine, and Licentiate in Physic of the University of Oxford; Physician to the Henley Dispensary, &c. . . . . . . 134

Opinions on the Causes and Effects of the Disease denominated Tic Douloureux; deduced from practical Observations of the supposed Origin, in Lateral Pressure, Distortion, or undue Contact of the Teeth, &c. &c. With Engravings and Cases. By CHARLES BEW, Surgeon-Dentist to his Majesty, and the Royal Household, &c. . . . . . . 145

An Essay on Curvatures and Diseases of the Spines, including all the Forms of Spinal Distortion, to which the Fothergillian Gold Medal was awarded by the Medical Society of London, and presented at a special General Meeting, on the 3d of May, 1824; with some Additions. By R. W. BAMPFIELD, Esq. one of the Surgeons of the Royal Metropolitan Infirmary for Children, &c. &c. . . . . . . . . 222, 299

Outlines of a System of Medico-Chirurgical Education; containing Illustrations of the Applications of Anatomy, Physiology, and other Sciences, to the principal practical Points in Medicine and Surgery. With coloured Plates. By THOMAS TURNER, Member of the Royal College of Surgeons 308

An Essay on Venereal Diseases, and the Uses and Abuses of Mercury in their Treatment. Illustrated by Drawings of the different Forms of Venereal Eruptions. Second Edition. By RICHARD CARMICHAEL, M.R.I.A. &c. &c. . . . . . . . . . 405, 510

On Medical Education; being a Review of a "Dialogue between the Author (Dr. PARIS,) and a Practitioner who is about to direct the medical Studies of his Son" . . . . . . . . . 418

An Account of the Disease lately prevalent at the General Penitentiary. By P. MERE LATHAM, M.D. Fellow of the Royal College of Physicians, &c. . . . . . . . . . . . . 489

Third Vindication of the General Penitentiary, &c. By GEORGE HOLFORD, M.P. . . . . . . . . . . . ib.

### FOREIGN.

L'Art de Prolonger la Vie de l'Homme. Par C. F. HUFELAND, premier Medecin de S. M. le Roi de Prusse; Chevalier de l'Ordre de l'Aigle rouge, de second Classe; Professeur de Medecine de l'Université de Berlin, &c. &c. Traduit de l'Allemand sur la seconde Edition. Par A. J. G. JOURDAIN, Docteur en Medecine de la Faculté de Paris . . 154

Observations Pathologiques propres a eclairer plusieurs points de Pathologie. Par F. LALLEMAND, Professeur de Clinique Chirurgicale a la Faculté de Montpellier . . . . . . . . . . 238

PAGE

Manuel d'Anatomie Générale, Descriptive et Pathologique, par J. F. Meckel, Professeur d'Anatomie à l'Université de Halle. Traduit de l'Allemand, et augmenté des Faits nouveaux dont la Science s'est enrichie jusqu'a ce Jour, par A. J. L. Jourdan, Membre des Académies Royales de Médecine de Paris . . . . . 246, 331, 423, 521

C. Wenzel on Diseases of the Spine; with eight copper-plates . . 318

### MISCELLANEOUS.

Proceedings in the University of Edinburgh . . . . . . 80

Surgeon and Apothecaries' Drug Company . . . . . . 82

Bills of Mortality . . . . . . . . . . 84

Notice respecting the Case of Anne Coulthurst . . . . 171

Regulations of the College of Edinburgh . . . . . . 344

Report of the Vaccine Establishment . . . . . . 347

Prizes of the Medical Society of London . . . . . . 346

Table of Admissions into the Small-Pox Hospital . . . . 348

Unpublished Manuscripts of Morgagni . . . . . . 437

### OBITUARY.

Dr. Cleverley . . . 84
Dr. R. Harrison . . . 172
R. Schetky, Esq. . . . 261
G. E. Ewbank, Esq. . . . 532

### MONTHLY CATALOGUE OF BOOKS.

Pages 85, 174, 261, 349, 533.

### METEOROLOGICAL TABLES.

Pages 86, 173, 262, 349, 442, 534.

### NOTICES TO CORRESPONDENTS.

Pages 86, 174, 262, 350, 442, 534.

### NAMES OF AUTHORS,

*Of whose Works, Observations, &c. either a detailed Account, or more or less general View, is given in*

### THE FIFTY-THIRD VOLUME

### OF THE LONDON MEDICAL AND PHYSICAL JOURNAL.

PAGE

**Anderson**, Mr. case of Irritative Fever . . . . . 162
**Andral**, M. on Rupture of the Spinal Marrow . . . . 435
**Antommarchi**, Dr. his Anatomical Plates . . . . 4

**Baker**, Dr. case of Disease of the Heart . . . . . 477
**Bartels**, Professor, Experiments upon decapitated Robbers . . 131
**Bayle**, Mr. on anomalous Gout . . . . . . 13
**Beck**, Dr. on Feigned Diseases . . . . . . 393
**Belcher**, Dr. on Purpura Hæmorrhagica . . . . . 176
**Benedict**, Dr. on Extirpation of a Tumor . . . . 25
**Blackett**, Mr. on an Instrument for compressing the Carotid Artery . 380
**Bouillaud**, M. connexion between the anterior Lobes of the Brain and the Tongue . . . . . . . . . 528
**Bougon**, M. on a Cicatrix in the Heart . . . . . 259
**Bush**, Mr. on a case of Twins . . . . . . 121
———— on the Fracture-Cradle . . . . . . 342

**Calderini**, Dr. on the Juice of the Euphorbia Lathyris . . . 530

**Cantu**, Dr. on Mercury in the Urine . . . . . 164

**Cayol**, M. on Affection of the Brain . . . . . ib.

**Chevalier**, Mr. on the Operation for Strangulated Hernia . . 467

**Children**, Mr. on an Acid in the Stomach . . . . 13

**Church**, Dr. on Ergot . . . . . . . . 18

**Civiale's** Lithontriptor . . . . . . . . 32

**Clever**, M. on Lithotomy . . . . . . . 31

**Cloquet**, M. on Acupuncturation as a cure in Ophthalmia . . 437

**Coqueteau**, M. on the Reproduction of the Crystalline Lens . . 529

**Collins**, Dr. remarks on Midwifery . . . . . 170

**Corban**, Dr. case of Fracture and Depression of Frontal Bone . . 182

**Crawford**, Dr. on the Semidecussation of the Optic Nerves . . 48

**Creaser**, Dr. on Amputation . . . . . . 31

**Davis**, Dr. on Pustules under the Tongue in Hydrophobia . . 163
**De Salle**, M. on Iodine in Gonorrhœa . . . . . 19
**Delpech**, M. on Extirpation of the Jaw . . . . . 26
**Dray**, Dr. on Goitre . . . . . . . . 49
**Dufaur**, M. on Oil of Turpentine in Rheumatism . . . . 18
**Dupau**, M. on a new species of Cinchona . . . . . 436
**Dutrochet**, M. on Muscular Contraction . . . . . 10
**Duvergie**, M. on an unnatural Growth of the Bones of the Cranium . 529
**Dewees**, Dr. on Acids in the Stomach . . . . . 530
———— on Deformities of the Pelvis . . . . . ib.

PAGE

Eccum, Dr. on Snuff-taking . . . . . . . . 166
Esquirol, M. case of Strangulation of the Intestines . . . 435

Fontanelle, M. Table of new Vegetable Principles . . . 33
Fox, Dr. on the use of the Stomach Syringe . . . . 208
Francini, Dr. on Absorption . . . . . . 9

Griffith, Mr. case of Perforation of the Stomach . . . . 287

Godman, Dr. method of preserving Bodies . . . . . 528

———— mode of preventing the ill Effects of Wounds in Dissection . 530

Guibourt, Mr. on the Oxydes and Sulphurets of Mercury . . . 127

Guthrie, Mr. remarks on Dr. Collins' Letter . . . . 384

Harrison, Dr. E. remarks upon cases of Spinal Disease . . . 443

Hayman, Dr. Extirpation of a Tumor . . . . . . 26

Henry, Mr. cases of Tic Douloureux . . . . . 474

Hottot, M. on preparing Morphia . . . . . . 168

Hufeland, M. on Blood-letting . . . . . . . 15

———— on Enlargement of the Brain . . . . 256

———— on the Disappearance of the Mammæ, caused by Iodine . 437

Husson, M. on Pomegranate Bark in Tænia . . . . 165

James, Dr. on Antimony and Ipecacuanha in Dyspepsia . . . 529
Jones, Mr. on the Pathology of Fever . . . . . 108, 289
———— on Sympathy . . . . . . . 204
———— on Gout . . . . . . . . 385
Jolly, M. case of Diseased Spine . . . . . . 257

Kinglake, Dr. on Purgative Medicine . . . . . 185
Knox, Dr. on the Lacteal System in the Testaceous Mammalia . . 9

Laennec, M. on the Treatment of Inflammation . . . . 17
Larkin, Mr. on Contagion . . . . . 263, 351, 448
Lassaigne, M. on detecting the Rust of Blood . . . . 532
Lauth, M. on the Lymphatic System . . . . . . 9
Lippi, Dr. his discovery of a Lymphatic Vessel . . . . 255
Lizars, Mr. on Extirpation of the Ovaria . . . . . 27

Mackenzie, Mr. on the Muscularity of Arteries . . . . 87

Magendie, M. on the sense of Smell . . . . . 528

———— on the Sensibility of the Nerves . . . . 434

———— on the Nerves . . . . . . . 4

Martinet, M. on Inflammation . . . . . . . 11

Marc, M. case of a Child crying in Utero . . . . . 260

Mondini, M. on the Eye . . . . . . . . 8

Morichini, Professor, on Croton Oil in Inflammation of the Bowels . 436

Nicholls, Dr. case of punctured Wound . . . . . 180

Nævus on Nævi . . . . . . . . . 166

North, Mr. on Convulsions in Children . . . . . 39, 284

Palotta, Galileo, on Pariglina . . . . . . . 343
Pelletan, M. on the Phenomena of Acupuncturation . . . 434
Pollock, Mr. on Mercurial Action in Syphilis . . . . 178
Prevost and Dumas, MM. on the Development of the Fœtal Heart . 11
Prout, Dr. on an Acid in the Stomach . . . . . 13

Ranking, Mr. on Neuralgia . . . . . . . 124

Recamier, M. case of Death from Acupuncturation . . . 531

Ridgway, Dr. on the Treatment of Ophthalmia . . . . 122

———— on administering Peruvian Bark . . . . 295

PAGE

ROCHOND, M. on Syphilis . . . . . . . 20

ROLANDO, M. on the Brain . . . . . . . 3

RUNGE, M. on the active principle of Belladonna . . . 169

SAVART, M. on the Membrana Tympani . . . . . 8

SCOUTETTEN, Dr. on the Pathology of the Peritoneum . . . 11

——————— on Enlargements of the Brain . . . 256

SERRES, M. on Comparative Anatomy . . . . . 1

SHATH, Mr. on fresh Vegetables in Fever . . . . . 165

SHAW, Mr. on Wounds received in Dissection . . . 369

——————— on the Inclined Plane . . . . . . 481

SMITH, Dr. GORDON, on Medical Evidence . . . . 213

ST. VINCENT, M. on Generation . . . . . . 10

STOKER, Dr. on the Humoral Pathology . . . . . 364

THOMAS, Mr. case of Rupture of the Heart . . . . 193

——————— case of a Child who swallowed a Nail . . . 480

THOMPSON, Dr. A. T. case of Fever from Dissection . . . 438

TREVIRANUS, Dr. on the Cochlea of Birds . . . . . 254

TULLY, Mr. on the Medical Topography of Malta . . . . 194

VANDEBURGH, Dr. case of Abscess of the Liver . . . . 42
VAUQUELIN and SEGELAS, MM. on Sugar in the Blood in Diabetes . 163
——————— on the active principle of Colocynth . 170
VENABLES, Dr. on Varioloid Disease . . . . . 461
VELPEAU, M. on an unusual appearance of the Blood . . . 435

WALLER, Mr. on Puerperal Irritability . . . . 116, 381
——————— on Gastrotomy . . . . . . 191
WALTHER, Professor, on Amputation at the Hip . . . . 21

YEATMAN, Mr. on the Treatment of Hernia . . . . . 54

